# Natural killer cells associated with SARS-CoV-2 viral RNA shedding, antibody response and mortality in COVID-19 patients

**DOI:** 10.1186/s40164-021-00199-1

**Published:** 2021-01-27

**Authors:** Changqian Bao, Xiandong Tao, Wei Cui, Yuanyuan Hao, Shuaike Zheng, Bin Yi, Tiewen Pan, Ken H. Young, Wenbin Qian

**Affiliations:** 1 Department of Hematology, The Second Affiliated Hospital, Zhejiang University School of Medicine, 310009 Hangzhou, China; 2 The Third Affiliated Hospital of Naval Medical University, 200438 Shanghai, China; 3 Wuhan Huoshenshan Hospital, 430100 Wuhan, China; 4 Department of Intensive Care Unit, The Second Affiliated Hospital, Zhejiang University School of Medicine, 310009 Hangzhou, China; 5 Department of Dentist, The Second Affiliated Hospital, Zhejiang University School of Medicine, 310009 Hangzhou, China; 6 Hematopathology Division, Department of Pathology, Duke University Medical Center, Duke University Cancer Center, Durham, NC USA; 7 National Clinical Research Center for Hematologic Diseases, The First Affiliated Hospital of Soochow University, Jiangsu 215006 Suzhou, China

**Keywords:** COVID-19, SARS-CoV-2, Natural killer cell, RNA viral shedding, Antibody response

## Abstract

Coronavirus disease 2019 (COVID-19) is a novel infectious viral disease caused by the severe acute respiratory syndrome coronavirus 2 (SARS-CoV-2). Two consecutively negative SARS-CoV-2 viral RNA test ( interval ≥ 24 hours), improved respiratory symptoms and obvious absorption of inflammation in pulmonary imaging are the discharge criteria for COVID-19 patients. The clearance profile of viral RNA in the upper respiratory tract specimens, including nasopharyngeal swab and/or oropharyngeal swabs, is related to innate immune cells such as Natural Killer cells. A total of 168 patients were included for the study. In this cohort, non-severe and severe groups showed significant differences in white blood cells, neutrophils, lymphocytes, basophils and platelets counts, as well as in infection related parameters such as CRP and serum cytokine IL-6. For lymphocyte subsets tests at admission, the severe group displayed significantly lower cell counts than the non-severe group. Higher counts of total T cells, CD4 + T cells, CD8 + T cells, and NK cells in peripheral blood showed a significant correlation with the shorter time taken to obtain the first negative viral RNA test and first positive IgM/ IgG antibody test. The number of B cells was only correlated with time to achieve the first positive IgM/IgG test. The count of NK cells was also correlated with a higher level of IgG antibody (*p* = 0.025). The lymphocytopenia group had a significantly worse survival rate (*p* = 0.022) and a longer duration (*p* = 0.023) of viral shedding than the normal lymphocyte count group. A lower NK cell count correlates the most with the worse survival rate (*p*<0.001) and a longer duration (*p*<0.001) of viral shedding. This study suggests the potential value of allo-Natural Killer cell therapy as an universal COVID-19 treatment strategy.

**To the editor,**

A novel coronavirus, severe acute respiratory syndrome coronavirus 2 (SARS-CoV-2), has spread globally since December 2019. Factors that affect the duration of viral shedding of SARS-CoV-2 [1] have not been fully declared yet. The association between lymphocyte subsets, including Natural Killer cell count, viral RNA shedding, and antibody response remain unclear. A total of 168 COVID-19 patients were retrospectively enrolled, followed and analyzed as methods indicated (Additional file 1: Methods) for this study (Additional file 2: Table S1). Non-severe and severe groups showed significant differences in white blood cells, neutrophils, lymphocytes, basophils and platelets counts, as well as in infection related parameters such as CRP and serum cytokine IL-6. The severe group had higher level of CRP and IL-6 than non-severe group. For lymphocyte subsets tests at admission, the severe group displayed significantly lower cell counts than the non-severe group (Additional file 3: Table S2). SARS-CoV-2 viral RNA detection and specific IgM and IgG tests were taken at least twice per week after admission. Higher counts of total T cells, CD4 + T cells, CD8 + T cells, and NK cells in peripheral blood showed a significant correlation with the shorter time taken to obtain the first negative viral RNA test and first positive IgM/ IgG antibody test.The severe group required a longer time to achieve the first positive IgM/IgG test (either IgM or IgG more than 10 IU/mL) (Additional file 4: Table S3). Inflammatory monocytes were reported to secret IL-6 and GM-CSF in COVID-19 patients,resulting in elevated IL-6 and other cytokines such as IFN-γ and TNF-α [2]. IL-6 mediated JAK/STAT signalling which could elicit cytokines release storm (CRS) and correlated with worse prognosis. According to our data, higher CRP and IL-6 had a significant correlation with a longer time taken to achieve the first viral RNA negative and IgM/IgG positive tests. The number of B cells was only correlated with time to achieve the first positive IgM/IgG test. The count of NK cells was also correlated with a higher level of IgG antibody (*p* = 0.025) (Additional file 5: Table S4 and Additional file 1: Additional material ). The lymphocytopenia (<1 × 10^9^/L) [3] group had a significantly worse survival rate (log-rank *p* = 0.022) and a longer duration (log-rank *p* = 0.023) of viral shedding than the normal lymphocytes count group. The NK cell lymphocytopenia (<0.1 × 10^9^/L) [4] group had a significantly worse survival rate (log-rank *p* < 0.001) and a longer duration (log-rank *p* < 0.001) of viral shedding (Fig. 1).

**Fig. 1:**
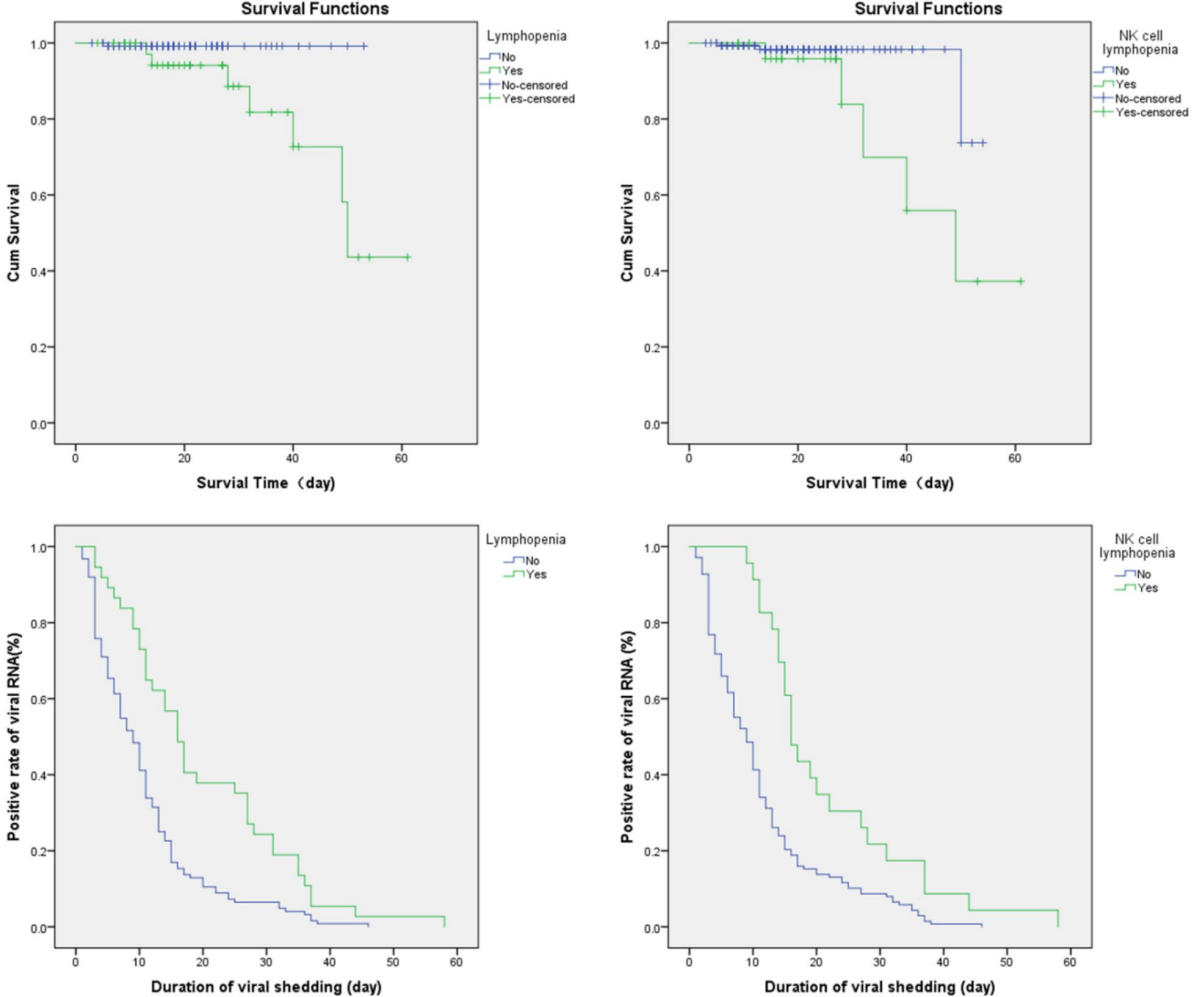
Survival and hazardous analysis. In lymphocytopenia subgroups analysis, *p* = 0.022 for survival and *p* = 0.023 for viral shedding duration. In NK cell lymphocytopenia *p* < 0.001 for survival and *p* < 0.001 for viral shedding duration

Both innate and adaptive immune responses are essential to control viral infections. NK cells, as a part of innate immune system, play an important role during acute viral infection [5]. The strength and duration of IgG after infection is the key point of immunity from SARS-CoV-2 [6]. NK cells have a direct killing effect on virus-infected cells through the killer-cell immunoglobulin-like receptors (KIR) [7], lysis-infected cells and releasing antigen. NK cells interact with dendritic cells [8] and may play a part in processes of antigen-presentation and adaptive immunity against SARS-CoV-2. NK cell counts could be critical to the IgG immunity for COVID-19. Engagement of CD16 on NK cells by antibody-coated virus-infected cells results in antibody-dependent cellular cytotoxicity [9]. The innate immune system serves as a frontline of clearance for coronavirus and regulates immune response. We hypotheses that NK cells can directly kill virus-infected cells through degranulation, receptor mediated apoptosis, and antibody-dependent cell-mediated cytotoxicity (ADCC) (Fig. 2) [10, 11]. Also, it has been proven that NK cells conduct active crosstalk with autologous dendritic cells (DC) through a process that requires NK cell-DC cell interaction and the secretion of specific cytokines [12]. This may explain why the NK cell count is related to a specific IgM/IgG antibody response. So far, there are four COVID-19 related NK cell clinical trials registered on Clinicaltrials.gov, including hematopoietic stem cell derived NK cells infusion (NCT04365101), NK Cells in combination with standard therapy (NCT04280224), IL-15 superagonist- and GM-CSF neutralizing scFv-secreting NKG2D-ACE2 CAR-NK cell therapy for COVID-19 patients (NCT04324996). Natural Killer cells do not cause GVHD, which provides an off-shelf anti-viral cell therapy. This study suggests the potential of Natural Killer cells as an universal COVID-19 treatment.

**Fig. 2:**
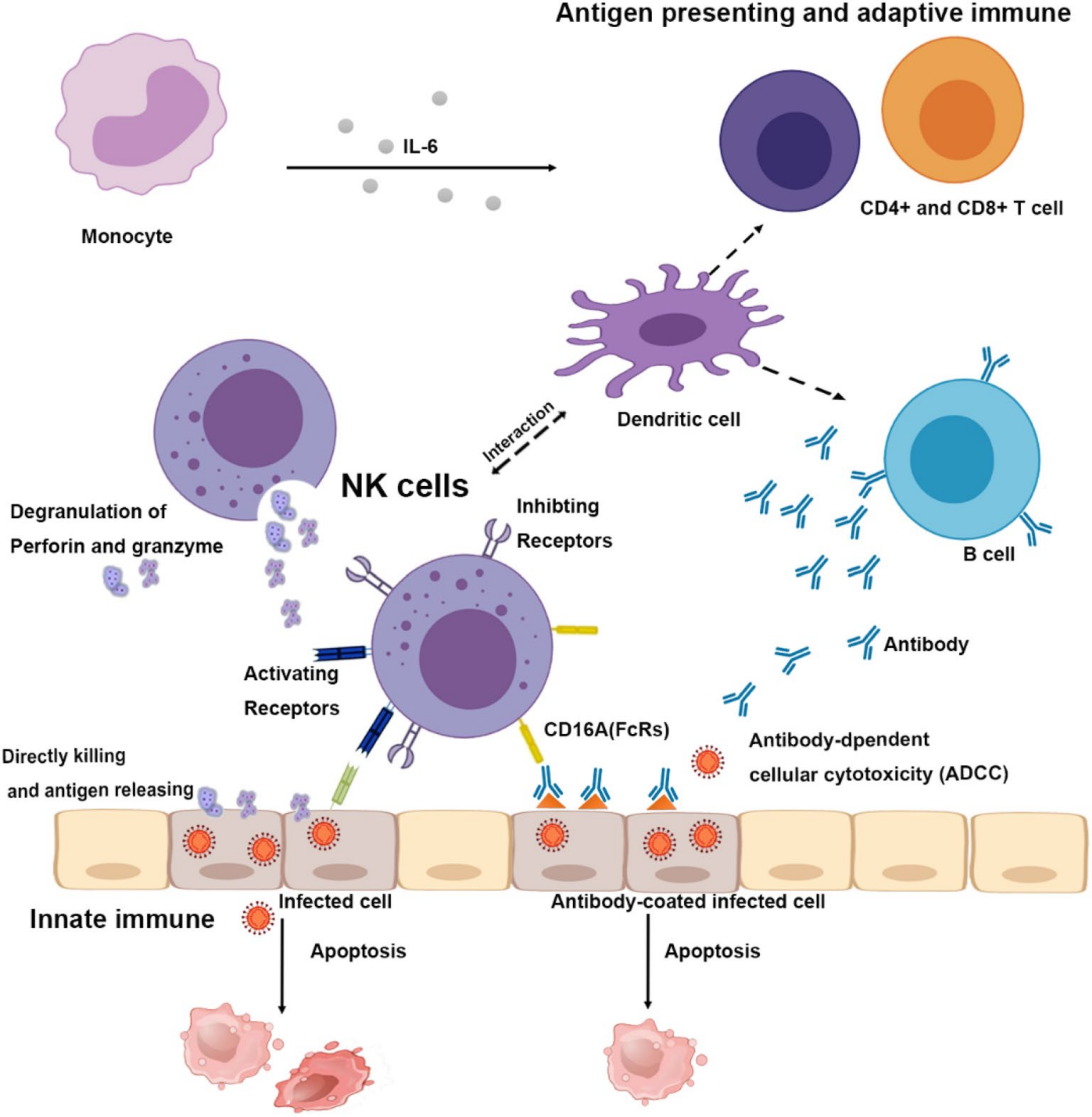
The innate and adaptive immune response to SARS-CoV-2 infection. Hypothesis of the innate and adaptive immune response to SARS-CoV-2 infection. NK cells have direct killing effect on SARS-CoV-2 infected cells, as well as participate in antibody-dependent cellular cytotoxicity. Through inhibiting and activating receptors, NK cells recognize infected cells. Once activated, the degranulation of NK cells induces the releasing of perforin and granzyme which directly lyse infected cells. NK cells express CD16A, which participates in antibody-dependent cellular cytotoxicity. Meanwhile, NK cells also interact with dendritic cells in antigen presenting process and affect adaptive immune response. Monocytes can secret IL-6 that acts as inflammatory cytokine, resulting in the activation of other immune cells

## Supplementary Information

**Additional file 1.** Additional material-Methods.

**Additional file 2: Table S1.** Clinical characteristics of patients.

**Additional file 3: Table S2.** Laboratoryexamination at admission.

**Additional file 4: Table S3.** Time to achieve the first SARS-CoV-2 nucleic acid negative test and the first positiveIgM/IgG test.

**Additional file 5: Table S4.** Correlation analysis.
