# Supplementary material for: Natural killer cells associated with SARS-CoV-2 viral RNA shedding, antibody response and mortality in COVID-19 patients
Source: Exp Hematol Oncol. 2021 Jan 27;10:5. doi: 10.1186/s40164-021-00199-1 (PMC7839286; doi:10.1186/s40164-021-00199-1)
Supplement: Supplementary file 1 — Additional file 1. Additional material-Methods. [file 40164_2021_199_MOESM1_ESM.docx]

**Supplemental material**

**Methods**

A total of 168 COVID-19 patients who were hospitalized in Huoshenshan hospital between Feb 10, 2020 and April 15, 2020 were retrospectively enrolled, followed and analyzed for this study. The enrollment criteria included testing positive for SARS-CoV-2 in the real-time reverse transcription PCR (real-time RT-PCR) and being diagnosed according to WHO interim guidance [1]. The study was conducted in accordance with the Declaration of Helsinki, and the protocol was approved by the Ethics Committee and institution research board of Huoshenshan hospital，the Third Affiliated Hospital of Naval Medical University and the Second Affiliated Hospital, Zhejiang University School of Medicine.

All patients had taken the required laboratory tests and chest computerized tomography (CT). All laboratory tests were performed in compliance with the clinical needs of patients. Detailed clinical symptom presentations, laboratory findings, which included complete blood count (CBC), serum interleukin-6 (IL-6), lymphocyte subsets, SARS-CoV-2 nucleic acid PCR analysis, IgM/IgG antibody test, and radiographic evaluation, were analyzed. Upon admission, we classified the patients into two subtypes, non-severe and severe, according to the severity of their diseases and the American Thoracic Society guidelines for community-acquired pneumonia [2]. Continuous variables were described as median and interquartile range (IQR). Correlations between quantitative variables were performed using Pearson correlation. Statistical analysis was done using Statistical Product and Service Solutions (SPSS) version 22.0.

1. Organization WH: **Clinical management of severe acute respiratory infection (SARI) when COVID-19 disease is suspected. Interim guidance.13 March 2020** *https://wwwwhoint/publications-detail/clinical-management-of-severe-acute-respiratory-infection-when-novel-coronavirus-(ncov)-infection-is-suspected*.

2. Metlay JP, Waterer GW, Long AC, Anzueto A, Brozek J, Crothers K, Cooley LA, Dean NC, Fine MJ, Flanders SA *et al*: **Diagnosis and Treatment of Adults with Community-acquired Pneumonia. An Official Clinical Practice Guideline of the American Thoracic Society and Infectious Diseases Society of America**. *American journal of respiratory and critical care medicine* 2019, **200**(7):e45-e67.
