# Supplementary material for: Natural killer cells associated with SARS-CoV-2 viral RNA shedding, antibody response and mortality in COVID-19 patients
Source: Exp Hematol Oncol. 2021 Jan 27;10:5. doi: 10.1186/s40164-021-00199-1 (PMC7839286; doi:10.1186/s40164-021-00199-1)
Supplement: Supplementary file 2 — Additional file 2: Table S1. Clinical characteristics of patients. [file 40164_2021_199_MOESM2_ESM.docx]

| Table 1 Clinical characteristics of patients. |  |  |
| --- | --- | --- |
| All patient(N=168) |  |  |
| **Gender** |  |  |
| Female | 87 | 51.8% |
| Male | 81 | 48.2% |
| **Age: Median（****interquartile range）** | 63 | (56-72) |
| **Disease Severity** |  |  |
| Non-severe | 101 | 60.1% |
| Severe | 67 | 39.9% |
| **Achieve SARS-CoV-2 Nucleic Acid negative test** |  |  |
| Yes | 161 | 95.8% |
| No | 7 | 4.2% |
| **Outcome** |  |  |
| Death | 8 | 4.8% |
| Survivor | 160 | 95.2% |
| Data are shown as n (%). |  |  |
