# Supplementary material for: Natural killer cells associated with SARS-CoV-2 viral RNA shedding, antibody response and mortality in COVID-19 patients
Source: Exp Hematol Oncol. 2021 Jan 27;10:5. doi: 10.1186/s40164-021-00199-1 (PMC7839286; doi:10.1186/s40164-021-00199-1)
Supplement: Supplementary file 3 — Additional file 3: Table S2. Laboratoryexamination at admission. [file 40164_2021_199_MOESM3_ESM.docx]

Table 2 Laboratory examination at admission.

|  | **Non-severe** | **Severe** |  |  |
| --- | --- | --- | --- | --- |
|  | **Median (interquartile range)** | **Median (interquartile range)** | ***P*** |  |
| **Blood Cell Counts** |  |  |  |  |
| White Blood Cells (3.5-9.5 x10^9^/L） | 6.400(5.400-8.095) | 5.100(4.100-6.900) | 0.004 |  |
| Neutrophils (1.8-6.3 x10^9^/L) | 4.030(2.940-5.320) | 3.340(2.270-4.550) | 0.027 |  |
| Lymphocytes (1.1-3.2 x10^9^/L) | 1.590(1.250-1.965) | 1.010(0.730-1.410) | 0.000 |  |
| Monocytes(0.1-0.6 x10^9^/L) | 0.470(0.390-0.605) | 0.400(0.300-0.570) | 0.150 |  |
| Eosinophils(0.02-0.52 x10^9^/L) | 0.140(0.080-0.235) | 0.100(0.060-0.170) | 0.055 |  |
| Basophils(0-0.06 x10^9^/L) | 0.020(0.020-0.035) | 0.020(0.010-0.030) | <0.001 |  |
| Hemoglobin(130-175 g/L) | 124.000(114.000-136.000) | 120.000(108.000-129.000) | 0.051 |  |
| Platelets (125-350 x10^9^/L) | 236.000(196.000-281.500) | 188.000(132.000-240.000) | <0.001 |  |
| **Infection related parameters** | |  |  |  |
| CRP(0-4mg/L) | 2.310(0.590-7.370) | 5.520(1.640-53.480) | 0.001 |  |
| **Serum Cytokine** |  |  |  |  |
| IL-6(pg/ml） | 1.500(1.500-5.055) | 5.330(2.460-43.610) | 0.018 |  |
| **Lymphocyte subtype** |  |  |  |  |
| Total T Cell（CD3+)% （52.86%-79.90%） | 68.600(62.150-75.500) | 72.100(64.800-76.900) | 0.164 |  |
| CD4+T Cell% (23.73%-50.37%) | 39.200(32.550-46.100) | 39.400(33.500-46.900) | 0.784 |  |
| CD8+T Cell % （13.73%-38.64%） | 24.400(18.100-29.850) | 25.300(20.300-31.700) | 0.257 |  |
| B Cell %（4.5%-18.1%） | 10.400(6.000-14.050) | 9.900(5.900-13.700) | 0.524 |  |
| NK Cell% (6.17%-32.67%） | 17.100(13.200-23.850) | 13.800(8.500-17.700) | 0.004 |  |
| Total T Cell Number(0.797-2.37 x10^9^/L) | 1.049(0.861-1.349) | 0.739(0.454-1.041) | <0.001 |  |
| CD4+T Cell Number (0.432-1.341 x10^9^/L) | 0.574(0.473-0.805) | 0.420(0.266-0.617) | <0.001 |  |
| CD8+ T Cell Number (0.238-1.075 x10^9^/L) | 0.374(0.261-0.522) | 0.242(0.170-0.402) | <0.001 |  |
| B Cell Number(0.086-0.594 x10^9^/L) | 0.159(0.090-0.236) | 0.094(0.050-0.160) | <0.001 |  |
| NK Cell Number(0.127-0.987 x10^9^/L) | 0.273(0.209-0.374) | 0.129(0.086-0.192) | <0.001 |  |

Data are median value (interquartile range).
