# Supplementary material for: Natural killer cells associated with SARS-CoV-2 viral RNA shedding, antibody response and mortality in COVID-19 patients
Source: Exp Hematol Oncol. 2021 Jan 27;10:5. doi: 10.1186/s40164-021-00199-1 (PMC7839286; doi:10.1186/s40164-021-00199-1)
Supplement: Supplementary file 4 — Additional file 4: Table S3. Time to achieve the first SARS-CoV-2 nucleic acid negative test and the first positiveIgM/IgG test. [file 40164_2021_199_MOESM4_ESM.docx]

Table 3 Time to achieve the first SARS-CoV-2 nucleic acid negative test and the first positive IgM/IgG test.

|  | **Non-severe** | | **Severe** | |  |
| --- | --- | --- | --- | --- | --- |
|  | **Median (interquartile range)** | | **Median (interquartile range)** | | ***P*** |
| **Time to achieve the first positve IgM/IgG (Day)** | 1.000(1.000-8.000) | | 2.000(1.000-15.000) | | 0.054 |
| IgM (IU/mL) | 31.820(10.810-79.745) | | 40.220(11.960-74.110) | | 0.604 |
| IgG (IU/mL) | 166.620(124.120-188.395) | | 159.060(88.190-191.850) | | 0.296 |
| **Time to achieve the first SARS-CoV-2 nucleic acid negative test (Day)** | 7.000(3.000-13.000) | | 13.500(9.250-27.000) | | <0.001 |
| Data are median value (interquartile range). | |  | |  | |
