# Supplementary material for: Natural killer cells associated with SARS-CoV-2 viral RNA shedding, antibody response and mortality in COVID-19 patients
Source: Exp Hematol Oncol. 2021 Jan 27;10:5. doi: 10.1186/s40164-021-00199-1 (PMC7839286; doi:10.1186/s40164-021-00199-1)
Supplement: Supplementary file 5 — Additional file 5: Table S4. Correlation analysis. [file 40164_2021_199_MOESM5_ESM.docx]

| Table 4 Correlation Analysis | |  |  |  |  |  |  | |  | |  |
| --- | --- | --- | --- | --- | --- | --- | --- | --- | --- | --- | --- |
|  |  | CRP | IL-6 | Total T Cell Number | CD4+T Cell Number | CD8+ T Cell Number | B Cell Number | NK Cell Number | |  |  |
| Time to achieve the first SARS-CoV-2 viral RNA negative test | Correlation | 0.313^**^ | 0.279^**^ | -0.315^**^ | -0.179^*^ | -0.339^**^ | -0.140 | -0.670^**^ | |  |  |
|  | Sig. (2-tailed) | <0.001 | <0.001 | <0.001 | 0.023 | <0.001 | 0.076 | <0.001 | |  |  |
| Time to achieve the first positive IgM/IgG | Correlation | 0.328^**^ | 0.335^**^ | -0.261^**^ | -0.176^*^ | -0.313^**^ | -0.167^*^ | -0.302^**^ | |  |  |
|  | Sig. (2-tailed) | <0.001 | <0.001 | 0.001 | 0.022 | <0.001 | 0.031 | <0.001 | |  |  |
| IgM level | Correlation | -0.036 | -0.019 | 0.058 | 0.125 | -0.017 | -0.027 | -0.030 | |  |  |
|  | Sig. (2-tailed) | 0.645 | 0.803 | 0.455 | 0.106 | 0.826 | 0.726 | 0.695 | |  |  |
| IgG level | Correlation | 0.015 | -0.013 | 0.066 | 0.042 | 0.102 | 0.008 | 0.173^*^ | |  |  |
|  | Sig. (2-tailed) | 0.848 | 0.867 | 0.397 | 0.586 | 0.189 | 0.922 | 0.025 | |  |  |
| * *P*＜ 0.05 ； ** *P*＜ 0.01； | |  |  |  |  |  |  | |  | |  |
